# The inhibitory effects of 7ND protein on osteoclast differentiation in apical periodontitis

**DOI:** 10.3389/fcimb.2025.1597604

**Published:** 2025-06-27

**Authors:** Zhang-Zhang Ji, Xiu-Min Xu, Yu-Lin Lu, Run-Zhen Zhang, Ying Zhang, Zhi-Hui Zou, Qi Xu

**Affiliations:** ^1^ Department of Stomatology, The Second Affiliated Hospital of Anhui Medical University, Hefei, China; ^2^ Department of Pediatric Dentistry, Hefei Stomatological Hospital, Hefei, China; ^3^ Department of Physiology, School of Basic Medical Sciences, Anhui Medical University, Hefei, Anhui, China

**Keywords:** monocyte chemoattractant protein-1, 7ND, osteoclasts, apical periodontitis, osteolysis

## Abstract

**Background:**

Apical periodontitis (AP) is a highly prevalent inflammatory condition that affects the tissue surrounding the apex of a tooth root. 7ND protein, a mutant form of monocyte chemoattractant protein-1 (MCP-1), functions as a dominant negative inhibitor of MCP-1. Previous studies have shown that 7ND protein can suppress osteoclast differentiation in peripheral blood mononuclear cells, suggesting its potential to prevent inflammatory bone destruction. However, whether 7ND protein can inhibit AP-induced osteolysis remains unknown.

**Methods:**

To investigate the effects of 7ND protein on osteoclast differentiation, we utilized RAW264.7 macrophage cells and an AP rat model. Western blotting analysis was employed to assess MCP-1 expression in RAW264.7 cells treated with 7ND protein. The impact of 7ND protein on osteoclast formation was evaluated both *in vitro* (using RAW264.7 cells) and *in vivo* (in AP rats). Additionally, X-ray imaging and micro-computed tomography were used to compare the lesion volume and area in the periapical regions of AP rats treated with 7ND protein versus those treated with PBS.

**Results:**

Western blotting analysis revealed that 7ND protein reduced MCP-1 expression in RAW264.7 macrophage cells without affecting their proliferation. Furthermore, 7ND protein significantly inhibited osteoclast formation in both RAW264.7 cells and AP rats. In AP rats treated with 7ND protein, X-ray imaging and micro-computed tomography demonstrated a significant decrease in lesion volume and area in the periapical regions compared to AP rats treated with PBS.

**Conclusion:**

Our study demonstrates that 7ND protein has the potential to inhibit osteoclast differentiation and reduce bone loss associated with apical periodontitis. These findings suggest that 7ND protein may serve as a valuable therapeutic option for the treatment of AP-related osteolysis.

## 1 Introduction

Apical periodontitis (AP) is a common inflammatory disease affecting the dental pulp and surrounding tissues, which is characterized by the destruction of alveolar bone and the formation of a periapical lesion (Li et al., 2024). It is commonly caused by dental pulp bacterial infection, which induces a complex interplay of immune cells, inflammatory mediators, bone-resorbing osteoclasts, and results in alveolar bone defect and tooth loss (Liu et al., 2012; Wang et al., 2023). AP can develop when microorganisms infect the pulp through entries provided by dental caries, trauma, and defective dental restorations (Austah et al., 2016; Wang et al., 2022; Li et al., 2023). Bacterial invasion into the pulp tissue leads to periapical lesions, which then initiate a chronic infection as well as an inflammatory process; this results in periapical lesions development (de Oliveira et al., 2017; Ricucci et al., 2021). The inflammatory process in endodontic-origin lesions incorporates various cell activation contributing to bone tissue destruction, such as endothelial cells, neutrophils, macrophages, lymphocytes, mesenchymal stem cells, as well as osteoclasts (de Oliveira et al., 2017; Fan et al., 2023). The presence of bacteria and the products they produce trigger pro-inflammatory cytokines production, so inducing as well as maintaining an inflammatory response (Zymovets et al., 2023; Yekani et al., 2025). Due to impaired immune function, alveolar bone loss and tissue healing are exacerbated as a result of periodontal infections, leading to the formation of periapical lesions, which are often associated with significant bone resorption (Ricucci et al., 2021; Cui et al., 2025). The management of AP-induced bone resorption is critical for preserving tooth function and maintaining oral health. Conventional treatments for AP, such as root canal therapy, aim to eliminate the source of infection and reduce inflammation; however, they may not always be sufficient to halt or reverse the bone resorption process (Fang et al., 2021; Lasica et al., 2024). Therefore, the development of novel therapeutic strategies targeting the osteoclastogenic pathway represents a promising approach to mitigate the bone destructive effects of AP.

During times of acute inflammation, monocyte chemoattractant protein-1 (MCP-1) is a major released inflammatory cytokine, which is responsible for monocytes’ recruitment and activation (Melgarejo et al., 2009; Hao et al., 2020). Moreover, MCP-1 significantly promotes multinuclear cell fusion into osteoclasts and increases tartrate-resistant acid phosphatase (TRAP)-positive multinuclear bone-resorbing osteoclasts’ number *in vitro* (Kim et al., 2005, 2006). Osteoclasts are crucial in the bone destruction observed in cases of apical periodontitis, while RANKL (receptor activator of nuclear factor-κB ligand) is the key cytokine that promotes osteoclastogenesis (Hong et al., 2024). During apical periodontitis, RANKL expression is upregulated in response to inflammatory stimuli, leading to an increase in osteoclast formation and bone resorption. Therefore, targeting RANKL-mediated osteoclastogenesis represents a promising therapeutic strategy for managing AP and its associated bone loss.

A mutant form of MCP-1, called 7ND protein, lacks amino acids 2 through 8 on the N-terminal, which acts as an MCP-1 dominant negative inhibitor (Quan et al., 2014; Mulholland et al., 2019). The 7ND protein effectively reduces MCP-1-induced a human monocytic cell line (THP-1 macrophages) migration *in vitro* (Yao et al., 2014). Moreover, 7ND protein treatment significantly suppressed osteolysis induced by wear particles *in vivo* (Keeney et al., 2013; Jiang et al., 2016). Recently, Luo et al. found that 7ND functioned *in vitro* for osteoclast differentiation inhibition as well as for bone invasion progression reduction by oral squamous cell carcinoma *in vivo* (Luo et al., 2018). These results indicate that 7ND has emerged as a potential inhibitor of RANKL-mediated osteoclastogenesis. They further found that 7ND effectively forms inactive heterodimers with wild-type MCP-1, thereby blocking the recruitment of monocytes and macrophages to inflammatory sites. This was evidenced by the finding that local delivery of 7ND could significantly reduce osteoclast differentiation as well as alleviate lipopolysaccharide-induced osteolysis (Long et al., 2020). However, the role of 7ND in the context of apical periodontitis and its specific effects on RANKL-mediated osteoclastogenesis have not been fully elucidated. Here, we aim to examine the effects of 7ND on osteoclasts differentiation from RAW264.7 cells. Then we will investigate whether 7ND could reduce AP-induced bone erosion in an experimental rat model.

## 2 Materials and methods

### 2.1 Reagents

Recombinant mouse cytokine of RANKL (mRANKL) was purchased from PeproTech (Cranbury, NJ). Dulbecco’s Modified Eagle Medium (DMEM), phosphate-buffered saline (PBS) without calcium and magnesium, penicillin-streptomycin solution, fetal bovine serum (FBS), and rhodamine-conjugated phalloidin were obtained from Grand Island Biological Company (Grand Island, NY). The TRAP staining kit was acquired from Sigma-Aldrich (St. Louis, MO), and the Cell Counting Kit-8 (CCK-8) reagent was purchased from Dojindo Laboratories (Kumamoto, Japan).

### 2.2 Cell lines and culture

RAW264.7 macrophage cell line was purchased from the American Type Culture Collection (Manassas, VA, USA). RAW264.7 cells were cultured in DMEM supplemented with 10% fetal bovine serum and antibiotics (100 U/mL penicillin G and 100 μg/mL streptomycin) at 37°C in an incubator with 5% CO_2_ and 20% O_2_.

### 2.3 Isolation, expression, and purification of the 7ND protein

The 7ND was cloned and subsequently purified using the previously described method. Simply, the 7ND gene was amplified using MCP-1-based primers (GenBank: S71513.1) and cloned into the pMCSG7 vector (NovoPro Bioscience Inc., Shanghai, China). The recombinant plasmid harboring the 7ND gene was transformed into BL21 (DE3) E. coli cells, which were cultured in LB medium (Boster Biological Technologies, Ltd., Wuhan, China) with ampicillin at 37°C until OD_600_ reached 0.8. Induction with IPTG (0.2 mmol/L) was carried out at 16°C for 20 hours. Cells were harvested, lysed, and clarified by centrifugation. The supernatant was applied to a Ni-NTA column pre-equilibrated with PBS, washed, and eluted with imidazole. After buffer exchange, the His-tag was removed using tobacco etch virus protease. The protein fractions were pooled, concentrated, and purified by size exclusion chromatography.

### 2.4 Cell viability assay

RAW264.7 cells were incubated for 1day in 96-well plates at a density of 1×10^5^ cells/well. Subsequently, the medium (DMEM supplemented with 10% FBS and 1% penicillin/streptomycin) was completely replaced, and 7ND was added at increasing concentrations (0 ng/mL, 25 ng/mL, 50 ng/mL, and 100 ng/mL). Following 24, 48, and 72 hours, 10 microliters of CCK-8 reagent were added to each well, and the plates were then incubated for 3 hours in the dark. The absorbance was measured at 450 nm using a microplate reader (BioTek, Swindon, UK). To clarify the normalization procedure, the data were indeed standardized relative to the untreated control group (0 ng/mL 7ND) to ensure consistency with conventional reporting standards. The absorbance values at 450 nm for each experimental group (25 ng/mL, 50 ng/mL, and 100 ng/mL 7ND) were divided by the mean absorbance of the corresponding time point in the untreated control group (0 ng/mL).

### 2.5 Western blot analysis

A RIPA lysis and extraction buffer (Cat. No. 89900, Thermo Fisher Scientific Inc.) was used to extract the total protein from RAW264.7 cells. The lysate was clarified by centrifugation at 12,000 × *g* for 20 minutes at 4°C. After separation on an SDS-PAGE gel (Bio-Rad Laboratories, Hercules, CA), the samples were subsequently transferred to polyvinylidene fluoride membranes and blocked with dry skimmed milk (5%) in Tris-buffer saline for one hour at room temperature. The membranes were then incubated overnight at 4°C with primary antibodies against MCP-1 and GAPDH (cat. no. ab8101 and ab8245; 1:10,000 dilution), rinsed twice with PBST, and then incubated for 1 hour at room temperature with the HRP-conjugated secondary antibodies (cat. no. STAR137P; 1:3,000). The protein bands were visualized and captured via VersaDoc-MP Imaging System (Bio-Rad Laboratories, Hercules, CA). MCP-1 protein levels were quantified using ImageJ by normalizing the integrated density of MCP-1 bands to GAPDH. Background subtraction was performed with a rolling ball radius of 50 pixels, and only bands within the linear detection range were analyzed. Data are presented as MCP-1/GAPDH ratios.

### 2.6 Osteoclast differentiation and identification

RAW264.7 cells were plated in 24-well plates with mRANKL (40 ng/mL) for osteoclast differentiation induction. The experimental groups were as follows: Group 1, RAW264.7 cells only; Group 2, RAW264.7 cells with mRANKL (40 ng/mL); Group 3, RAW264.7 cells with mRANKL (40 ng/mL) plus 7ND (25 ng/mL). Both medium and all cytokines had been changed out every 3 days for 10 days, with osteoclasts identified as follows.

Cells of RAW264.7 first underwent 20 min 4% paraformaldehyde fixation then TRAP staining was performed as per manufacturer’s guides. Cells that were positive for the TRAP and contained three or more nuclei were regarded as multinucleated osteoclasts. In order to stain the actin cytoskeleton, RAW264.7 cells were rinsed 3 times with PBS, permeabilized for 10 minutes with 0.2% Triton X-100 in PBS, then stained with fluorescent phalloidin (1:200) for 20 minutes at room temperature before being observed by a fluorescence microscopy (Zeiss, Germany). The F-actin rings were defined as closed, circular F-actin structures at the cell periphery. Four random fields were selected and counted by two independent evaluators to determine both osteoclasts as well as F-actins numbers.

### 2.7 Animal model of AP and treatment

The animal experiments conducted in accordance with (ARRIVE) guidelines and laboratory animal care protocols approved by the Anhui Medical University Ethics Committee (approval number: LLSC20200801). Eighteen male Wistar rats (average weight of 210 g) were acquired from the Experimental Animal Center of Anhui Province maintained under temperature (20°C to 22°C), humidity (55% to 60%) with 12 h light/dark cycles. Rats were randomly assigned to three groups: Blank control group (Blank + PBS), AP control group (AP + PBS), and AP + 7ND treatment group (AP + 7ND). While the rats were undergoing ketamine anesthesia (90 mg/kg, intraperitoneal), a periapical lesion was induced by exposing right mandibular pulp first molar pulp via occlusion, as described previously (Xiao et al., 2015). On day 28 after lesion induction, six rats from each group were sacrificed. In rats of the AP + 7ND treatment group and the AP control group, 7ND or PBS was injected into right mandibular first molar periapical tissue every 2 days until day 28. The dosage of 7ND (3 μg/100 μl PBS) treatment for the AP model was determined based on prior studies (Luo et al., 2018; Long et al., 2020).

### 2.8 High-resolution X-ray imaging and micro-computed tomography analysis

To analyze rats’ bone intensity distribution of periapical lesions, high-resolution X-ray images were taken using a digital radiography system. The light dark region around the right mandibular first molar distal root apex indicates fairly low bone density; this area was assessed via Image-Pro Plus 6.0 (Media Cybernetics, Silver Spring, MD). All mandibles underwent scan by a micro-CT scanner (microCT µCT50, Scanco Medical AG, Zurich, Switzerland), at 8 W, 70 kV, 114 µA, 360° rotations, and 20 µm pixel size. Then the reconstructed 3D images were converted into graphic images. By two independent assessors, region of interest resorption areas in the right mandibular bones were delineated by Adobe Photoshop (Adobe Systems, Inc., San Jose, CA). Periapical region volume was measured as previously described (Xiao et al., 2015).

### 2.9 Histologic evaluation of osteolysis

All mandibles underwent fixation with 4% paraformaldehyde for 1 day, after which the soft tissue was detached. The remaining bones were decalcified for 3 weeks with 10% ethylenediaminetetraacetic acid (pH 7.4), then dehydrated and embedded in paraffin. Sections were sectioned at 5-µm thickness by a microtome, including the 7ND injection site. The slides were stained with TRAP in accordance with the instructions provided by the manufacturer. Two separate assessors carried out an analysis of osteoclasts numbers that were TRAP positive.

### 2.10 Statistical analysis

Data are expressed as mean ± SEM and evaluated for normal distribution by Shapiro–Wilk test. The differences among groups were tested by one-way analysis of variance test followed by a *post-hoc* Bonferroni correction. Statistical significance was determined by a P-value of 0.05 in all cases.

## 3 Results

### 3.1 7ND protein decreased MCP-1 amount but not affected the proliferation of RAW264.7 cells

**Figure 1:**
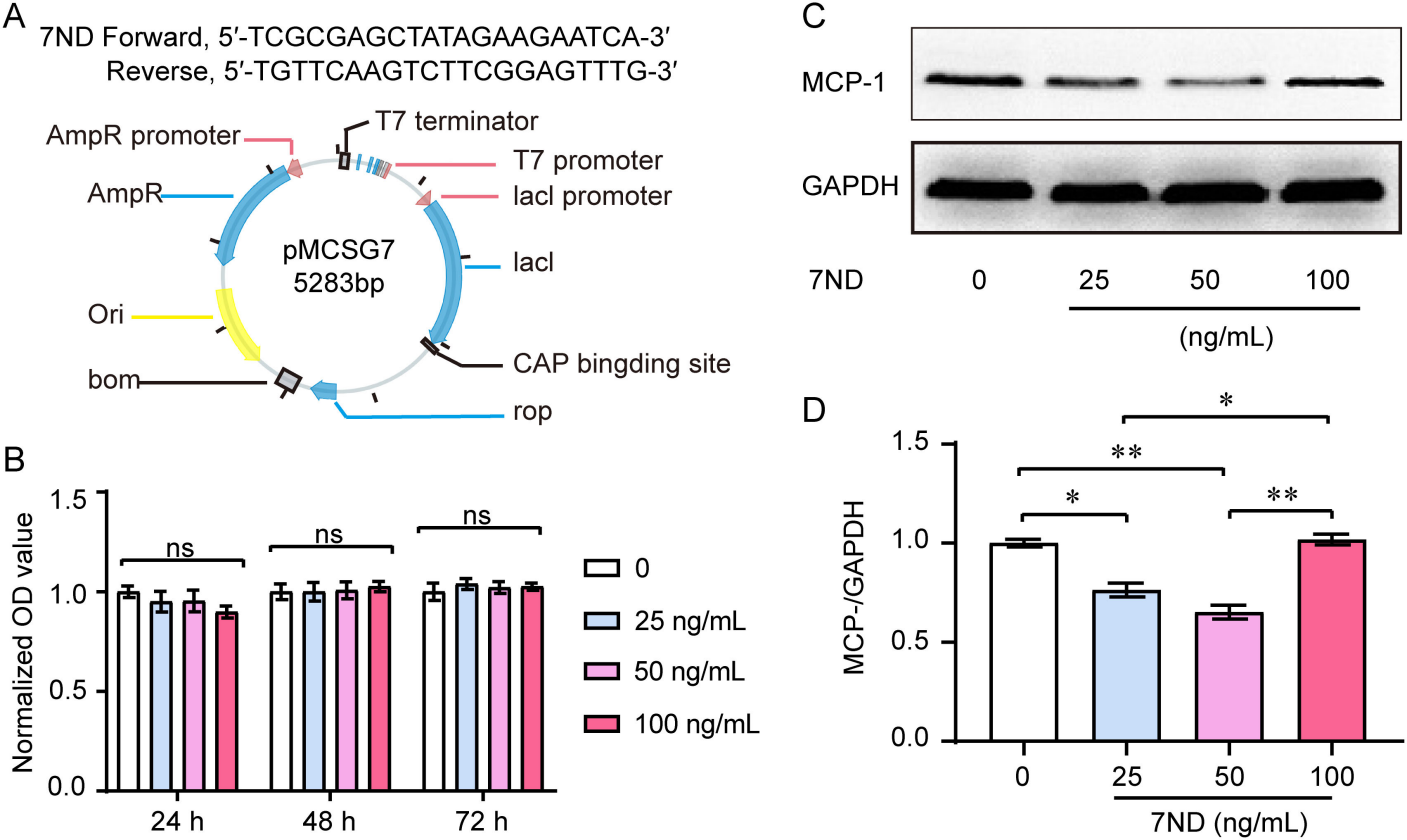
Effect of 7ND on cell viability and MCP-1 levels in RAW264.7 cells. **(A)** The 7ND gene was cloned into the pMCSG7 vector. **(B)** A CCK-8 assay showed that 7ND had no significant effect on the proliferation of RAW264.7 cells over 1 to 3 days as concentration of 7ND was increased (0 ng/mL, 25 ng/mL, 50 ng/mL, 100 ng/mL). Data are presented as the viability relative to untreated cells (0 ng/mL 7ND) and are displayed as the mean ± SEM. **(C)** Representative blots show the expression of MCP-1 in the absence or presence of 7ND treatment. **(D)** Western blotting results confirmed that the amount of MCP-1 in RAW264.7 cells was reduced after treatment with 7ND at concentrations of 25 ng/mL and 50 ng/mL. * *P <*0.05, ** *P* < 0.01 compared to all test groups, n = 6. ns, not significant.

The 7ND gene was engineered, cloned into the pMCSG7 vector, expressed, and the resulting 7ND protein was purified (**Figure 1A**). A CCK-8 assay was used to evaluate proliferation of RAW264.7 cells with or without 7ND protein. We found that the 7ND protein did not affect the proliferation of RAW264.7 cells over a period of 1 to 3 days as the concentrations of 7ND were increased (0 ng/mL, 25 ng/mL, 50 ng/mL, and 100 ng/mL, **Figure 1B**). The results of western blotting showed that 7ND protein reduced the amount of MCP-1 in RAW264.7 cells (**Figure 1C**). Concentrations of 25 ng/mL and 50 ng/mL of 7ND could efficiently reduce the protein amount of MCP-1 in RAW264.7 cells (F _(3, 20)_ = 36.45, *P* < 0.01). However, the higher concentration of 7ND (100 ng/mL) showed no significant effect on MCP-1 expression (**Figure 1D**). These results suggest that RAW264.7 cells express MCP-1, and its negative inhibitor, 7ND reduces the expression of MCP-1 without affect the proliferation of RAW264.7 cells.

### 3.2 7ND protein inhibited osteoclast differentiation from RAW264.7 cells

**Figure 2:**
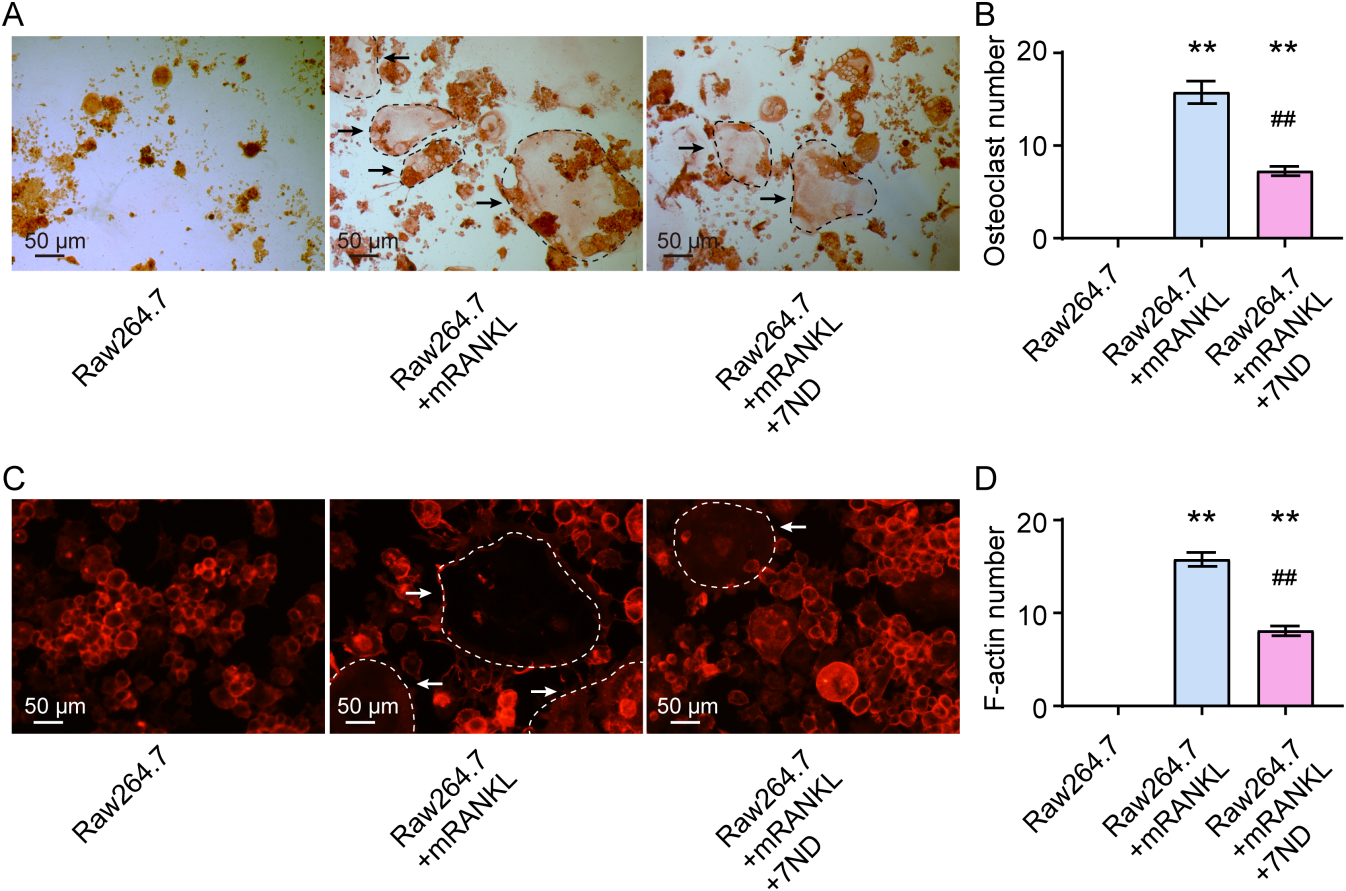
7ND protein inhibits osteoclast differentiation in RAW264.7 cells. **(A)** Representative TRAP staining images demonstrate the effect of 7ND protein administration on osteoclast differentiation. The black arrows indicate TRAP-positive cells (outlined by black dashed circles in the images). **(B)** Quantification of osteoclast numbers in four randomly selected fields. **(C)** Immunofluorescence staining reveals phalloidin for F-actin rings (outlined by white dashed circles in the images). The white arrows indicate cells with dense F-actin. **(D)** Quantification of F-actin ring numbers in four randomly selected fields. ** *P* < 0.01 compared with RAW264.7; ^##^ *P* < 0.01 compared with RAW264.7 + mRNAKL. n = 6.

When RAW264.7 cells were inducted to differentiate into osteoclasts by mRANKL for 14 days, the effect of 7ND protein at a concentration of 25 ng/mL on osteoclast differentiation was tested. The representative images of TRAP staining are shown in **Figure 2A**. The results revealed that significantly fewer osteoclasts were formed in the presence of 7ND treatment, while significantly more osteoclasts were formed in the presence of mRANKL alone (F _(2, 15)_ = 108.1, *P* < 0.01, **Figure 2B**). Immunofluorescence staining with phalloidin showed similar findings, with significantly less F-actin staining of osteoclasts observed in the presence of 7ND. No osteoclasts were observed in the blank control group, which contained RAW264.7 cells only (**Figure 2C**). Quantification of F-actin staining results is shown in **Figure 2D** (F _(2, 15)_ = 222.6, *P* < 0.01). These results indicated that the osteoclast differentiation from RAW264.7 cells was inhibited by 7ND protein.

### 3.3 7ND protein reduced periapical lesions in rats

**Figure 3:**
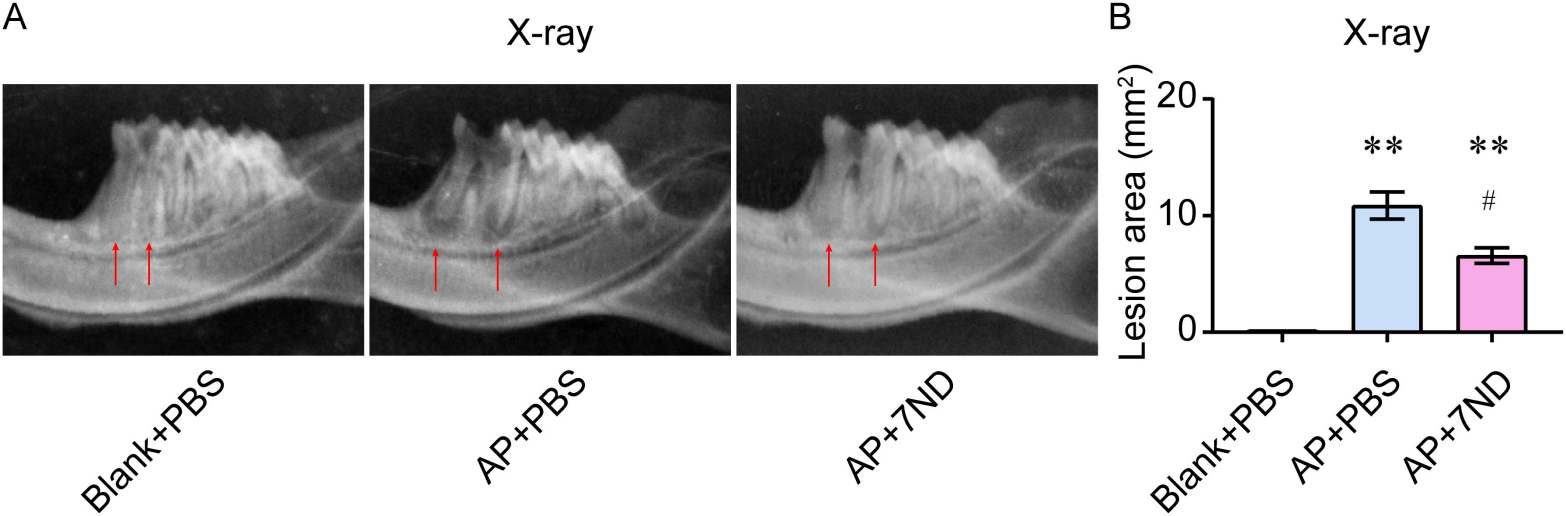
7ND protein reduces AP-induced periapical lesions in rats. **(A)** Representative X-ray images show periapical lesions in experimental rats. In the Blank + PBS group, no periapical lesions were observed around the distal root apex of the right mandibular first molar (arrow). In the AP + PBS group, periapical lesions of increasing size (arrow) were observed. In the AP + 7ND group, the size of the periapical lesions was reduced (arrow). **(B)** Quantification of the lesion area in the periapical regions of the Blank + PBS, AP + PBS and AP+7ND groups. ** *P* < 0.01 compared with Blank + PBS group; ^#^ *P* < 0.05 compared with AP + PBS group; n = 6.

The area and volume of the periapical region of right mandibular first molar was detected using high-resolution X-ray imaging. In the Blank control rats, there were no periapical lesions observed around the distal apex of the right mandibular first molar (**Figure 3A**). However, in the AP rats, periapical lesions of increasing size were observed. After the 7ND treatment, the size of periapical lesions was reduced in the AP+7ND rats. The quantitative analysis of lesion area showed that the size of the periapical region in the AP + 7ND group was significantly smaller than that in the AP + PBS group (F _(2, 15)_ = 299.3, *P* < 0.01, **Figure 3B**). These results suggest that the lesion area of the periapical regions was significantly decreased after the administration of 7ND in AP rats.

### 3.4 7ND protein reduced osteolysis in rats

**Figure 4:**
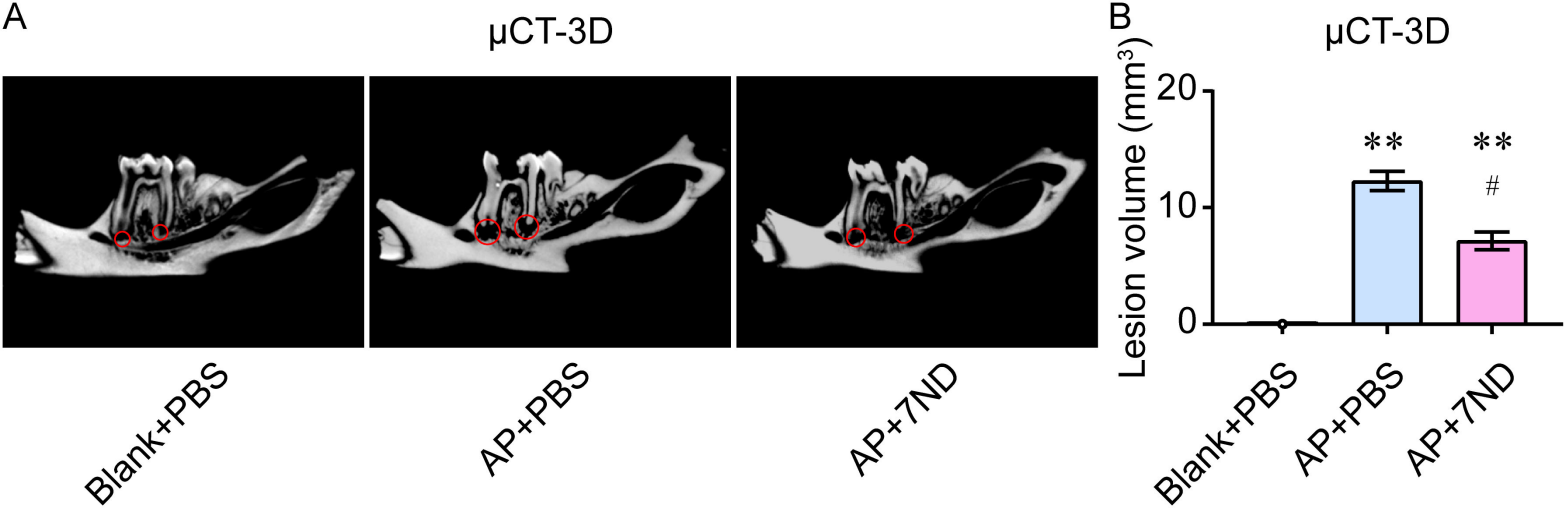
7ND treatment reduces AP-induced osteolysis in the calvaria. **(A)** Representative micro-CT images show the osteolysis in rats. Notably, bone defects in the mandible bone of the periapical region of the right mandibular first molar were less prevalent in the AP + 7ND group compared to the AP + PBS group (red circle). **(B)** Quantification of lesion volume in the periapical regions of the Blank + PBS, AP + PBS and AP + 7ND groups. ** *P* < 0.01 compared with Blank + PBS group; ^#^ *P* < 0.05 compared with AP + PBS group; n = 6.

The lesion volume of the periapical regions was used to quantify the degree of bone loss in rats. Fewer defects were observed in the periapical region of the right mandibular first molar in the mandible bone in the AP + 7ND group compared to the AP + PBS group, as assessed by micro-CT imaging (**Figure 4A**). Following 7ND treatment, the lesion volume in the periapical regions was significantly reduced in the AP+7ND rats compared to the AP + PBS rats (F _(2, 15)_ = 545.1, *P* < 0.01, **Figure 4B**). These results suggest that 7ND has a protective effect on the periapical lesions and osteolysis in AP rats.

### 3.5 7ND reduced the number of osteoclasts in the bone resorption lacunae

**Figure 5:**
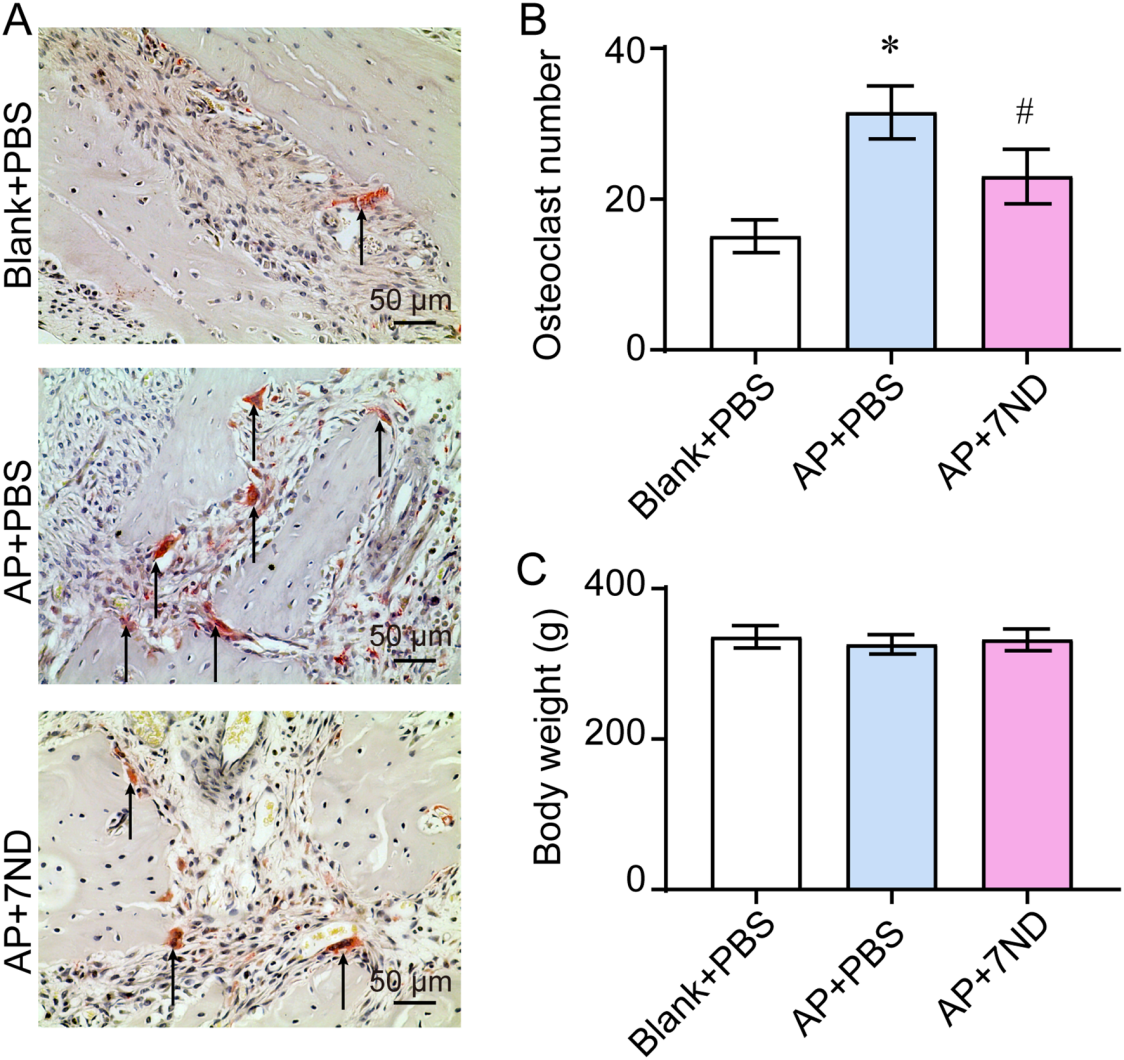
7ND reduces the number of osteoclasts in bone resorption lacunae. **(A)** TRAP staining was employed to locate and count osteoclasts accumulating in the resorption lacunae (black arrow). **(B)** Quantification of osteoclast numbers in the Blank + PBS, AP + PBS and AP + 7ND groups. **(C)** The body weight of rats over the 28 days of establishing the animal model in the Blank + PBS, AP + PBS and AP + 7ND groups. * *P* < 0.05, compared with Blank + PBS group; ^#^ *P* < 0.05 compared with AP + PBS group; n = 6.

To evaluate the effect of 7ND on osteoclasts in bone resorption lacunae, TRAP staining was performed to locate and subsequently quantify the osteoclasts accumulating in these lacunae (**Figure 5A**). The number of osteoclasts significantly increased in the AP + PBS group and decreased following 7ND treatment (F _(2, 15)_ = 39.75, *P* < 0.01, **Figure 5B**). In addition, body weight was monitored to assess the health status of rats during the experimental procedure. There were no significant differences in body weight between the Blank + PBS, AP + PBS and AP + 7ND groups over the 28-day period of establishing the animal model (F _(2, 15)_ = 0.77, *P* = 0.48, **Figure 5C**). These results demonstrate that osteoclasts are more abundant in AP rats and that 7ND protein reduce the number of osteoclasts in bone resorption lacunae.

## 4 Discussion

**Figure 6:**
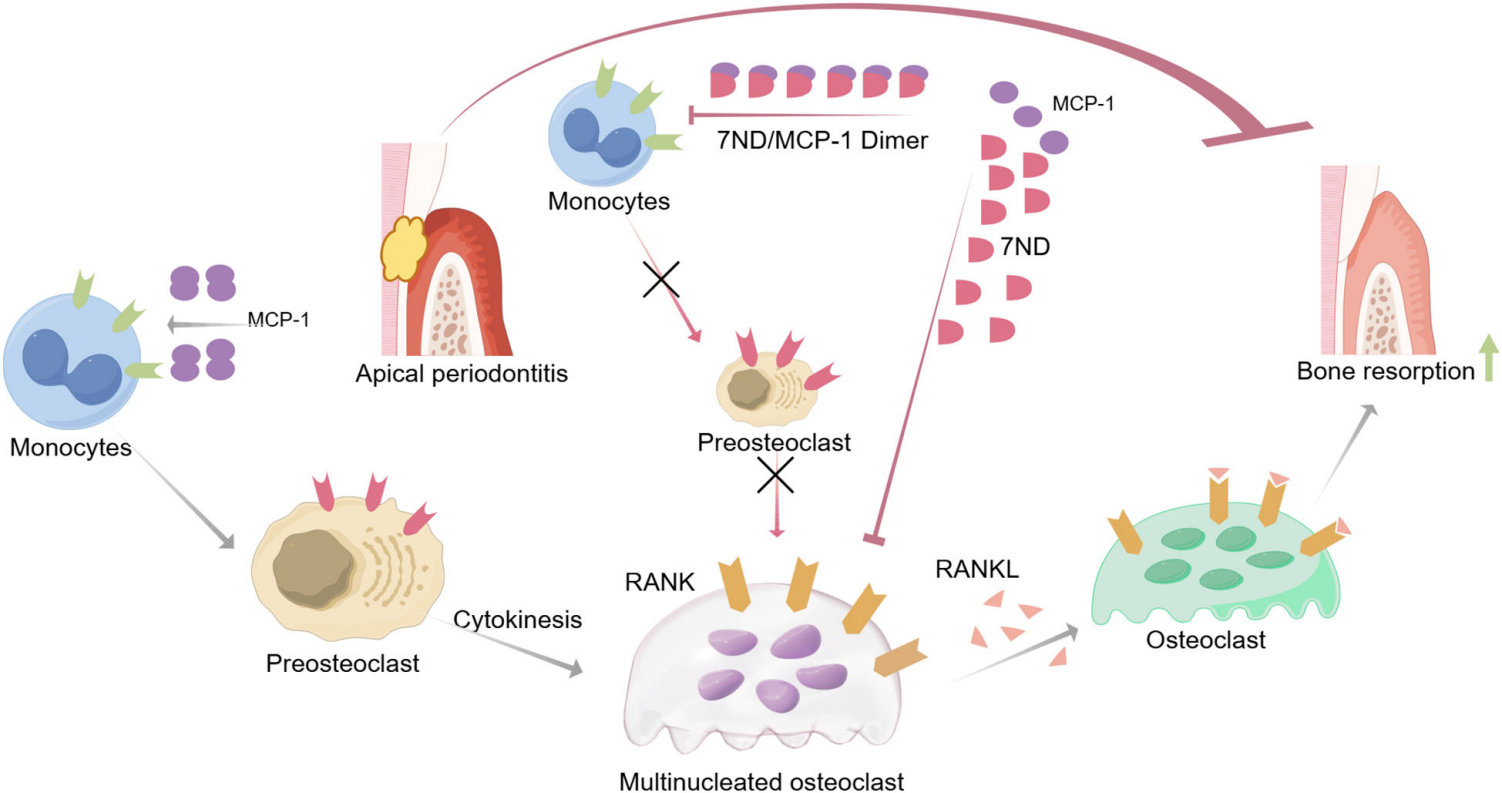
Schematic illustrating the inhibitory mechanisms of 7ND protein in attenuating apical periodontitis-associated bone resorption. MCP-1 facilitates monocyte recruitment to inflammatory sites and promotes their differentiation into osteoclast precursor cells. RANKL binding to RANK receptors on precursor cells triggers their fusion into multinucleated osteoclasts, initiating bone resorption. The 7ND protein exerts therapeutic effects through: MCP-1 sequestration via heterodimer formation, which disrupts chemotactic signaling and limits monocyte infiltration/osteoclast precursor expansion; and indirect suppression of osteoclastogenesis by reducing precursor cell availability. This sequential inhibitory strategy ultimately mitigates pathological bone destruction.

The present study has demonstrated that 7ND effectively inhibits RANKL-mediated osteoclastogenesis in RAW264.7 cells. Moreover, the results from the animal experiments provide further evidence of the therapeutic potential of 7ND. Specifically, the observed reductions in alveolar bone resorption and osteoclast numbers in the AP + 7ND group compared to the AP + PBS group suggest that 7ND may be effective in mitigating the bone destructive effects of apical periodontitis (**Figure 6**).

The significance of managing AP-induced bone resorption cannot be overstated, as it not only affects the integrity of the dental arch but also has broader implications for oral health and quality of life (Zhou et al., 2024). Alveolar bone loss, if left untreated, can lead to tooth mobility, eventual tooth loss, and impaired masticatory function (Marcello-MaChado et al., 2017; Kononen et al., 2019). Furthermore, the inflammatory process associated with AP can contribute to systemic inflammation, which has been linked to various systemic diseases, including cardiovascular disease and diabetes (Poornima et al., 2021; Li et al., 2025).

MCP-1, a member of the chemokine (C-C motif) family, plays an essential role in the processing of inflammation by functioning as a potent chemoattractant and activator for monocytes, macrophages, and osteoclast precursors. C-C motif chemokine receptor-2 (CCR2) serves as the primary receptor for MCP-1, and their interaction constitutes a crucial signaling axis that regulates the recruitment and activation of immune cells in various tissues, including bone (Singh et al., 2021). MCP-1 is presented in periapical granuloma samples from humans, as evidenced by immunohistochemical staining (Kabashima et al., 2001). Double immunofluorescence staining reveals that some MCP-1-positive cells also express CCR2, suggesting an interaction between MCP-1 and CCR2 in mediating inflammation and osteoclastogenesis (Liu et al., 2014). Upon injury or infection, the interaction between MCP-1 and CCR2 activates the signal transduction pathway, facilitating the migration and infiltration of macrophages, monocytes, microglia, and memory T lymphocytes in various diseases, including apical diseases (Salcedo et al., 2000; Silva et al., 2005).

Furthermore, MCP-1/CCR2 signaling plays a crucial role in bone remodeling and homeostasis by recruiting osteoclast precursors to the bone surface, where they differentiate into mature osteoclasts, enhancing bone resorption. Additionally, this signaling pathway contributes to the recruitment and activation of inflammatory monocytes and macrophages in periodontal disease, which can either differentiate into osteoclasts or stimulate osteoclastogenesis through RANKL production (Hong et al., 2024).

In the present study, we demonstrated that the expression of MCP-1, a crucial mediator of chemotaxis and inflammation, is constitutively expressed in RAW264.7 cells. A potent negative inhibitor for MCP-1, 7ND protein- a mutant MCP-1 lacking the first 2–8 amino acids at its N-terminus was found to effectively inhibit this expression in RAW264.7 cells. Specifically, the 7ND protein inhibited MCP-1 expression at concentrations of 25 ng/mL and 50 ng/mL, but not at 100 ng/mL. This observed effect may be due to the RAW264.7 cells developing compensatory or feedback mechanisms in response to the intermediate concentrations of the inhibitor. At the higher concentration of 100 ng/mL, the inhibiting effect of 7ND may be overridden, or the higher concentration may lead to non-specific binding of the inhibitor to other cellular components, potentially interfering with its ability to specifically inhibit MCP-1 expression. Consistent with previous findings, the 7ND protein and MCP-1 form a heterodimer that interacts with the CCR2 receptor, completely blocking the monocyte chemotaxis mediated by MCP-1 signal transduction (Quan et al., 2014; Jiang et al., 2016).

We further investigated the role of 7ND protein in osteoclast differentiation. RANKL was used to induce the osteoclast differentiation in RAW264.7 cells, and we found that treatment with 7ND significantly reduced osteoclast formation. In experiments where peripheral blood mononuclear cells were cultured in the presence of varying concentrations of 7ND, it was also observed that 7ND inhibited osteoclast differentiation without affecting cell proliferation, indicating that 7ND protein can effectively interfere with osteoclast differentiation (Long et al., 2020). This inhibition is achieved by blocking the MCP-1 chemokine action, which is crucial for the recruitment and migration of monocytes that eventually differentiate into osteoclasts. In addition, the 7ND protein efficiently prevented RAW264.7 macrophage cells from differentiating into osteoclasts without influencing cell proliferation. Given the possible coexistence and interaction of MCP-1 and RANKL in apical periodontitis, 7ND may indirectly affect RANKL-induced osteoclast differentiation by inhibiting MCP-1 signaling, although the specific mechanism of this indirect impact still needs further research (Long et al., 2020).

Although specific gene expression data related to 7ND’s effect on osteoclastogenesis are not widely available, it is plausible to infer that 7ND may regulate genes involved in the osteoclast differentiation pathway. In our study, we observed that 7ND protein, by inhibiting MCP-1, significantly reduced osteoclast formation both *in vitro* and *in vivo*. However, it is important to note that in the RAW264.7 cell experiments, the cells are already present in the culture environment, and thus, the chemotactic effect of MCP-1 is not a confounding factor. The inhibition of osteoclastogenesis by 7ND in this setting is likely due to its direct effects on osteoclast precursor differentiation, rather than its influence on cell recruitment. Furthermore, previous studies have shown that interfering with MCP-1 expression or inhibiting CCR2 could partially abolish SAK-HV-induced migration in RAW264.7 cells, indicating that MCP-1 plays a crucial role in cell migration (Chen et al., 2018). In line with our findings, administration of 7ND protein also significantly decreased the MCP-1-induced migration of THP-1 macrophage cells in a dose-dependent manner and reduced chemotactic effects caused by conditioned media from RAW264.7 cells (Yao et al., 2014). Additionally, previous studies have shown that the 7ND protein binds to MCP-1 without inducing cell activation, dramatically reducing inflammatory cytokines release without causing any significant adverse effects (Zhang and Rollins, 1995). Collectively, these promising results indicate the importance of MCP-1 and its signaling pathway in regulating immune cell recruitment, migration, and osteoclast differentiation, and highlight the potential therapeutic applications of targeting this pathway in diseases such as apical periodontitis and periodontal disease.

On the basis of 7ND’s inhibitory effects on osteoclast differentiation *in vitro*, we conducted an *in vivo* study using an animal model of AP. Through X-ray imaging besides micro-CT imaging, we confirmed that 7ND protein injection effectively mitigated AP-induced bone loss in rats. The treatment with 7ND protein significantly decreased osteolysis and preserved natural bone morphology. Histological investigations further revealed that 7ND administration successful lowered the total number of osteoclasts in AP rats. Consistent with these findings, previous studies have demonstrated the involvement of MCP-1 in inflammatory and resorptive processes in the periapical area, with distinct temporal patterns and cellular distributions. Both MCP-1 and TRAP-positive cells are observed in bone resorption lacune during AP progression. MCP-1-positive cells are distributed both centrally and peripherally in the periapical area, with their count correlating significantly with lesion size. Notably, MCP-1 upregulation is also observed in periodontitis patients at both the serum and tissue levels, with significantly elevated serum MCP-1 levels in periodontitis patients compared to healthy controls, as well as higher MCP-1 mRNA and protein expression in periodontitis tissues relative to controls (Gupta et al., 2013; Bostrom et al., 2015). Several pioneer studies have shown that 7ND therapy significantly reduced osteolysis induced by orthopedic implants wear particles (Jiang et al., 2016; Nabeshima et al., 2017; Luo et al., 2018). Wear particles raised the release of pro-inflammatory chemokines number, including MCP-1 (Sul et al., 2012; Yao et al., 2014; Suzuki et al., 2020; Zhu et al., 2021).Consistently, cellular studies have also demonstrated that RANKL treatment significantly increases MCP-1 in human colony forming unit-granulocyte macrophage precursors, while 7ND protein treatment blocks the induction of calcium signaling activator calmodulin 1, transcription factors JUN and NFATc2, dramatically inhibiting osteoclast differentiation (Morrison et al., 2014). These findings suggest that blockage of MCP-1 signaling at an early stage of inflammation-mediated stimulation may inhibit osteoclast differentiation induced by apical periodontitis.

Based on previous findings, it is plausible that 7ND’s therapeutic effect in AP is mediated through its inhibition of MCP-1 signaling. Additionally, 7ND’s antagonist activity on CCR2, a receptor for chemokines like C-C Motif Chemokine Ligand 2 (CCL2), suggests that it could potentially block the migration of CCR2-positive cells, such as monocytes and macrophages, towards these chemokines. Although the specific influence of 7ND on the migration of CCR2-positive cells in periapical lesions has not been directly addressed, its antagonist activity on CCR2 implies a potential modulation of the immune response associated with periapical lesions. However, further investigations are needed to reveal the precise molecular mechanisms of 7ND protein function on osteoclast differentiation and to explore its potential therapeutic applications in the treatment of periapical lesions.

## 5 Conclusions

The current study demonstrates 7ND protein’s therapeutic potential in inhibiting RANKL-mediated osteoclastogenesis and reducing alveolar bone resorption in apical periodontitis. Our results obtained from both cell experiments and animal models suggest that 7ND represents a promising therapeutic candidate for the treatment of apical periodontitis and related osteolytic disorders. Future research will conduct rigorous preclinical studies to optimize 7ND protein regimens, assess long-term safety, efficacy, and validate translational applications in subsequent clinical trials.

## Data Availability

The original contributions presented in the study are included in the article/**Supplementary Material.** Further inquiries can be directed to the corresponding authors.
