# Supplementary material for: The inhibitory effects of 7ND protein on osteoclast differentiation in apical periodontitis
Source: Front Cell Infect Microbiol. 2025 Jun 27;15:1597604. doi: 10.3389/fcimb.2025.1597604 (PMC12245798; doi:10.3389/fcimb.2025.1597604)

The Inhibitory Effects of 7ND Protein on Osteoclast Differentiation in Apical Periodontitis

Zhang-Zhang Ji[1†*], Xiu-Min Xu[2†], Yu-Lin Lu[3], Run-Zhen Zhang[1], Ying Zhang[1], Zhi-Hui Zou[3], Qi Xu[3]*

[1]Department of Stomatology, The Second Affiliated Hospital of Anhui Medical University, Hefei, China;

[2]Department of Pediatric Dentistry, Hefei Stomatological Hospital, Hefei, China;

[3]Department of Physiology, School of Basic Medical Sciences, Anhui Medical University, Hefei, Anhui, China

*** Correspondence**:

Qi Xu

qixu@ahmu.edu.cn

Zhang-Zhang Ji

jzz0407@qq.com

[†] These authors contributed equally to this work.

During the western blotting, the blots were cut prior to hybridize with antibodies.

FIGURE S1. Blots for GAPDH (~36 KDa)

7ND 100 50 25 0 (ng/mL) 0 25 50 100 (ng/mL)

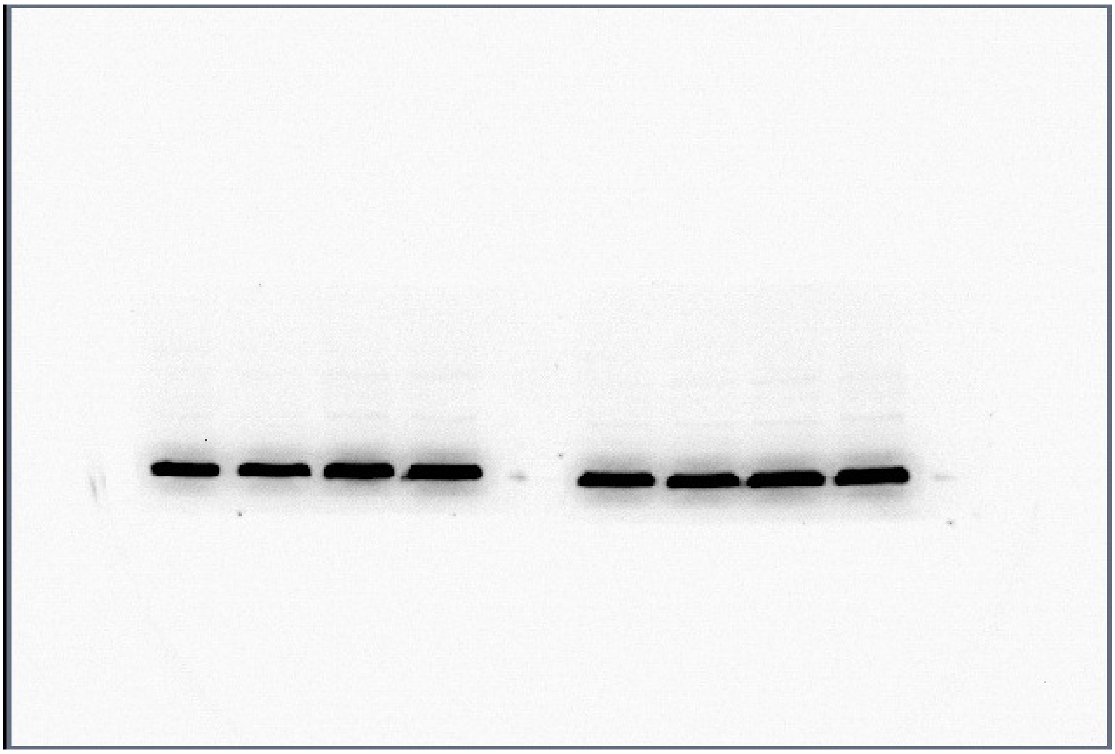

FIGURE S2. Blots for MCP-1 (~12 KDa)

7ND 100 50 25 0 (ng/mL) 0 25 50 100 (ng/mL)

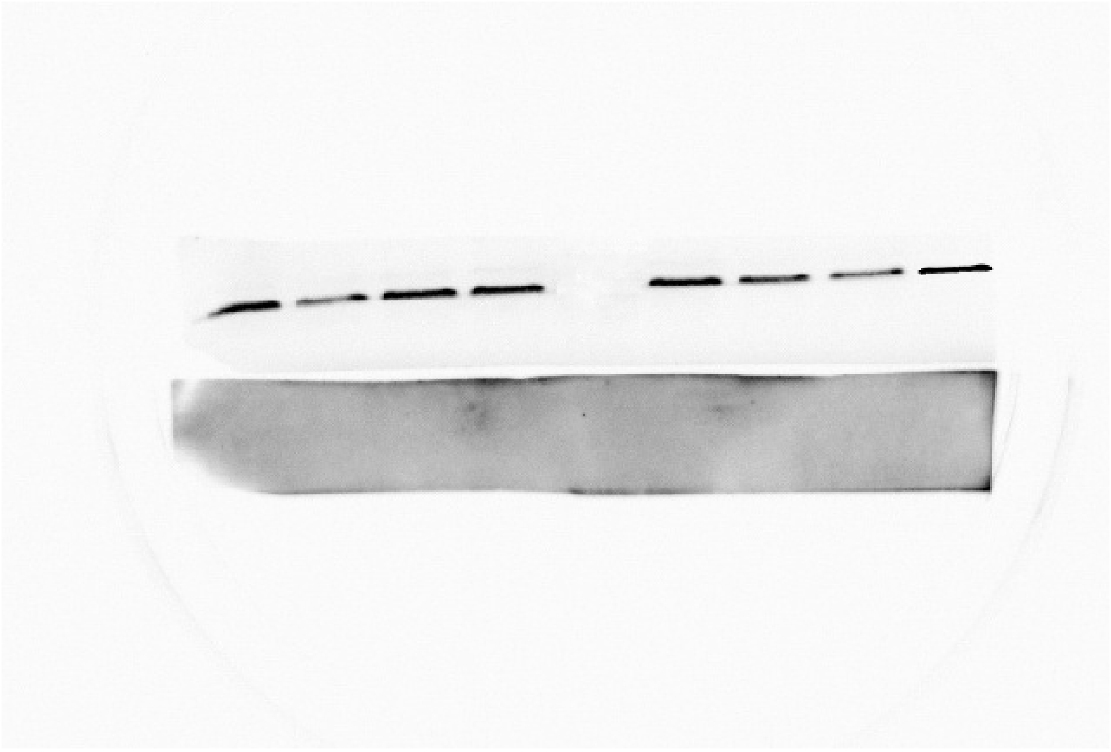

Supplement: Supplementary file 1 [file DataSheet1.pdf]
