# Supplementary figures and images for: The inhibitory effects of 7ND protein on osteoclast differentiation in apical periodontitis
Source: Front Cell Infect Microbiol. 2025 Jun 27;15:1597604. doi: 10.3389/fcimb.2025.1597604 (PMC12245798; doi:10.3389/fcimb.2025.1597604)

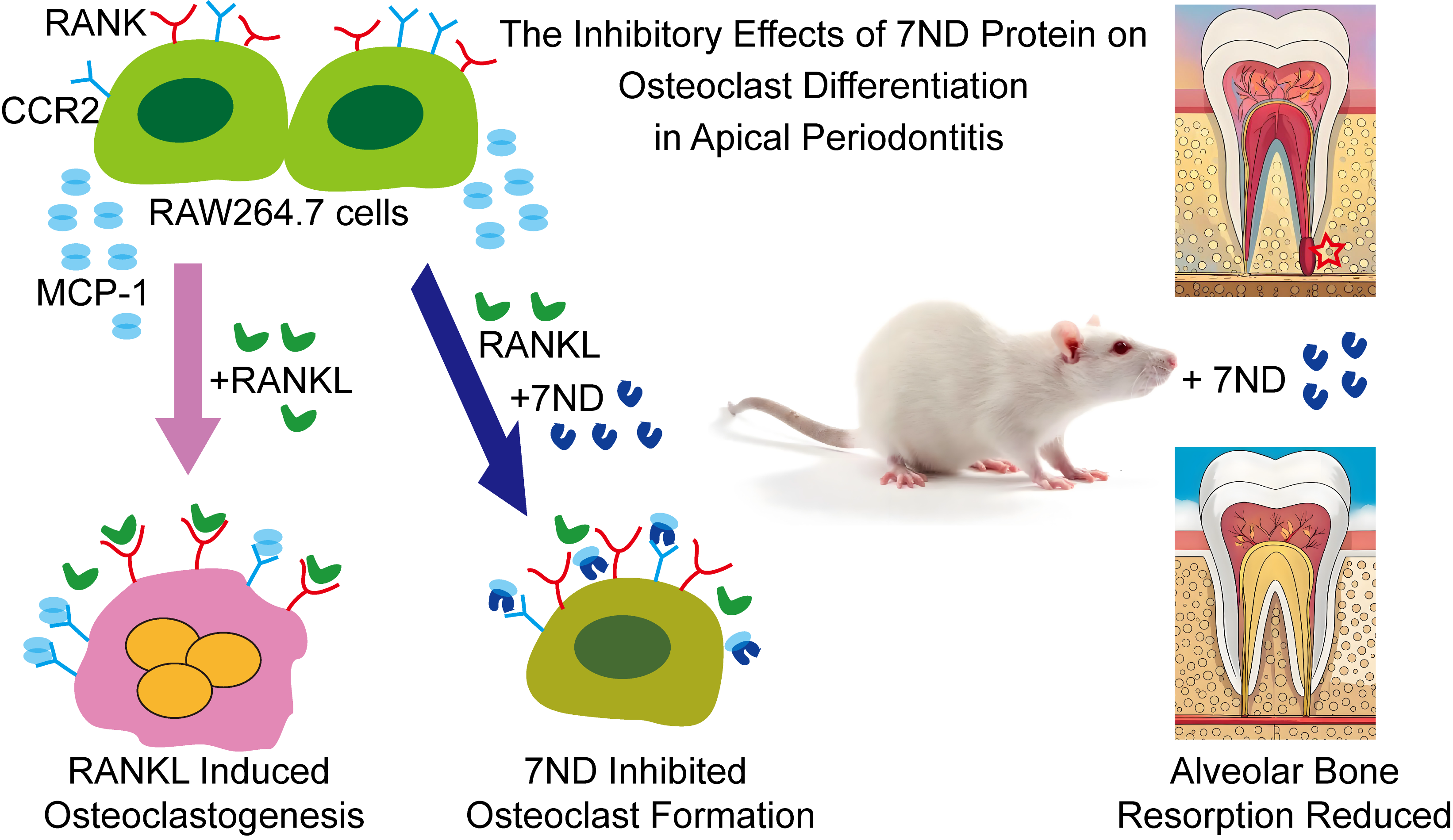

Supplement: Supplementary file 2 [file Image1.tif]
